# Effects of Graded Dietary Inclusion Level of Full-Fat *Hermetia illucens* Prepupae Meal in Practical Diets for Rainbow Trout (*Oncorhynchus mykiss*)

**DOI:** 10.3390/ani9050251

**Published:** 2019-05-17

**Authors:** Gloriana Cardinaletti, Basilio Randazzo, Maria Messina, Matteo Zarantoniello, Elisabetta Giorgini, Andrea Zimbelli, Leonardo Bruni, Giuliana Parisi, Ike Olivotto, Francesca Tulli

**Affiliations:** 1 Department of Agricultural, Food, Environmental and Animal Science, University of Udine, 33100 Udine, Italy; maria.messina@uniud.it (M.M.); francesca.tulli@uniud.it (F.T.); 2 Department of Life and Environmental Sciences, Polytechnic University of Marche, 60131 Ancona, Italy; matteo.zarantoniello@gmail.com (M.Z.); e.giorgini@staff.univpm.it (E.G.); andrea.zimbelli@gmail.com (A.Z.); i.olivotto@univpm.it (I.O.); 3 Department of Agriculture, Food, Environment and Forestry, University of Florence, 50144 Florence, Italy; leonardo.bruni@unifi.it (L.B.); giuliana.parisi@unifi.it (G.P.)

**Keywords:** black soldier fly, feed formulation, alternative proteins, growth metrics, FTIRI spectroscopy, gastrointestinal health, plasma metabolite

## Abstract

**Simple Summary:**

The sustainability of fish production is mainly driven by the protein source used in aquafeeds. In conventional fish feed, protein sources are mostly vegetable ingredients and fishmeal. The present study explored the potential use of full-fat *Hermetia illucens* prepupae meal (H) replacing 0% (H0), 25% (H25), and 50% (H50) conventional ingredients in practical diets for rainbow trout. No significant differences in growth were observed in all experimental groups, while in fish fed the H50 diet both hepatic and intestinal alterations were detected. In addition, in the same fish group, genes related to stress and immune-response were significantly up-regulated. The results obtained so far highlighted an overall physiological adaptation of fish to the dietary manipulation, suggesting an adverse effect of full-fat H at the highest inclusion level.

**Abstract:**

This study investigated the effects of dietary inclusion levels of full-fat *Hermetia illucens* prepupae meal (H) on growth and gastrointestinal integrity in rainbow trout (*Oncorhynchus mykiss*). A 98-day study was conducted using triplicate groups of trout (initial body weight, 137 ± 10.5 g) kept in 1-m^3^ tanks in a flow-through well water system. Three dietary treatments were prepared: one based on fishmeal and purified protein-rich vegetable ingredients (H0), and two experimental diets including graded levels of H meal (25% and 50%, referred to as H25 and H50, respectively). At the end of the feeding trial, no differences were observed in growth performance and plasma metabolite levels, with the biometric data confirmed by the liver expression of the genes involved in somatic growth regulation (*igf1* and *mstn1a*). In the H50 group, a three-fold up regulation of liver *hsp70* was observed. An activation of the stress/immune response (*il-10*, *tnf-α*, and *tlr-*5) was observed in medium intestine in the H25 and H50 groups (*p* < 0.05) together with a villi length reduction detected through histological analyses. Liver histology and Fourier Transform Infrared Imaging (FTIRI) spectroscopy highlighted an increase in lipid deposition. These findings suggest that caution should be taken into account when 50% replacement of conventional ingredients with H is selected.

## 1. Introduction

One of the most critical issues that threatens the sustainability and further growth of intensive aquaculture of carnivorous species is its dependency on fishmeal (FM) and fish oil (FO) in aquafeed [1]. Thus, alternative ingredients are needed to promote a sustainable aquaculture production while improving fish growth and health performance [2]. Over the last two decades, research efforts have focused on the reduction of the dietary inclusion levels of FM and FO by replacement with plant protein sources and vegetable oils, which are readily available on the feed market and cost-effective [1]. However, even if plant protein-based diets provided good results in some aquatic species, they presented some disadvantages for fish welfare and are often in direct competition with human nutrition [3,4,5], increasing pressure for the search of alternative and nutritional strategies to improve their utilization [3]. Some alternatives include animal feedstuffs, comprising both non-ruminant slaughterhouse by-products (derived from processed animal proteins, PAPs) and insect meals [6,7]. Insect meals show several advantages compared to conventional PAPs (poultry meal, hydrolysed poultry feathers, blood meal), as insects grow and reproduce quickly and easily on low-quality organic products [7]. Insect meals have a low ecological footprint [8], high feed conversion efficiency [9], and, together with PAPs, can reasonably foster a circular bio-economy.

In July 2017, the European Commission allowed the use of seven insect species as processed animal protein for aquaculture (Regulation 2017/893/EC, 2017). Although insect nutrient composition is dependent on taxonomic group, rearing substrate, and technological process, it usually shows high protein content (60–80% dry matter basis) with a well-balanced essential amino acid profile [10] and valuable lipid, mineral, and vitamin content [11,12]. These nutritional properties allows insects to be considered as a valuable alternative protein source, especially for carnivorous fish species, and several reviews on this topic are now available [11,12,13].

Among the different insect species considered for possible use in fish feeds [12,13], the dipteran *Hermetia illucens*, also known as black soldier fly (BSF), seems to be the most promising in that high quality standard industrial mass-rearing techniques already exist. The protein and lipid content of *H. illucens* meal (H) is variable depending on the processing technology and growth substrate. Generally, on dry matter basis, the defatted *H. illucens* meal protein and lipid contents range from 47.2 to 51.8% and from 11.8 to 14.8%, respectively [14,15]; whereas the protein and lipid contents reported for full-fat *H. illucens* meal are 36.2 and 18.0% [11], respectively. A major drawback in using *H. illucens* meal as an ingredient in aquafeeds is its lack of long-chain polyunsaturated fatty acids (LC-PUFAs) such as both the eicosapentaenoic (EPA, 20:5n-3) and docosahexaenoic (DHA, 22:6n-3) acids. The insect meal is rich in linoleic acid (LA, 18:2n-6), as well as saturated (SFAs) and monounsaturated (MUFA) fatty acids. The poor LC-PUFA content may be reflected in a low EPA and DHA content in the edible portion of fish (fillet), thus affecting the nutritional value for consumers. However, the great plasticity of *H. illucens* lipid composition, recently demonstrated by rearing the larvae on n-3 enriched substrates such as fish offal [16] or seaweeds [17], contributes to continuing interest in this species.

Despite the nutritional value of *H. illucens* meal (H), its successful inclusion level in aquafeeds depends on the fish species and the characteristics of insect derivatives [13]. Newton et al. [18] reported similar weight gain for channel catfish (*Ictalurus punctatus*) fingerlings fed diets containing up to 30% full-fat *H. illucens* prepupae meal. St-Hilaire et al. [19] did not find any negative effects on rainbow trout (*Oncorhynchus mykiss*) growth performance when a 15% full-fat *H. illucens* prepupae meal was included in the diet. The growth performance of trout fed diets containing 25% and 50% (dry matter basis) of *H. illucens* meal from prepupae reared on fish by-product enriched substrates showed results similar to those of fish fed the control FM-based diet [20]. Replacing up to 50% of FM with partially defatted-BSF larvae resulted in growth performance, body indices, and gut morphology similar to those of rainbow trout [21].

An additional controversial problem related to the inclusion of insect meal in aquafeeds is the presence of chitin [12]. Chitin was thought to play a role in shaping the gut microbial community [22] and to have positive effects on the innate immune response at moderate inclusion level in the diet (25–50 mg kg^−1^ chitin) [23]. However, when higher inclusion levels are used, the same macromolecule can adversely affect nutrient digestibility and uptake in fish [14,21,24] and negatively affects the intestinal mucosa integrity in amphibians [25].

Adequate nutrition is essential to guarantee fish health and digestive capacity; the morphological changes associated with the digestive system (liver and intestinal tract) are usually taken into account when alternative ingredients are considered in aquafeeds. In this regard, investigations of digestive system function of salmonids have been performed using histological [21,26,27,28], transcriptomic [29], proteomic [30], and molecular [31] approaches. Fourier Transform Infrared Imaging (FTIRI) spectroscopy, a fast, new and label-free inexpensive technique, is becoming more widely used. FTIRI allows researchers to obtain important biochemical information on the composition of biological samples through the macromolecule structures identification (lipids, proteins, carbohydrates, and nucleic acids) using the same sample at the same time. This method has recently been applied to the macromolecular characterization of trout and zebrafish (*Danio rerio*) intestine [32,33], gilthead seabream (*Sparus aurata*) liver [34], and clownfish (*Amphiprion ocellaris*) liver and intestine [35]. In the aforementioned studies, FTIRI highlighted that zebrafish fed insect meal-based diets resulted in a change of the intestinal mucosa macromolecular composition, showing an increase in the relative quantity of protein and fatty acid with longer alkyl chains [33]. On the other hand, the liver macromolecular composition of clownfish fed increasing levels of defatted *H. illucens* larvae meal [35] resulted in a lipid and protein decrease and an increase in glycogen. To the best of our knowledge, no similar information is available yet on trout fed diets including full-fat insect meal.

Coupled with the evaluation of gastrointestinal morphology and/or macromolecular composition, the relationship between dietary changes and metabolic profile, cytokine production [25], potential induction of inflammation [36,37] and stress biomarkers, may represent an useful tool for the overall fish welfare status evaluation.

The freshwater species of most interest to Italian aquaculture is rainbow trout (*Oncorhynchus mykiss*), with a volume of around 35,000 tons, which corresponds to 24.5% of the total national production [38]. In the present study, the effect of graded inclusion level of full-fat *Hermetia illucens* meal in a practical diet for this species was evaluated by assessing some growth indexes, plasma metabolites, and gastrointestinal health and stress biomarkers, using a multidisciplinary approach. The dietary inclusion levels of full-fat H meal were based on previous studies of insect meal [19,20] or other alternative vegetable protein sources [39,40,41,42] substitution. The hypothesis considered whether or not the full-fat H meal was a suitable alternative protein ingredient in a practical diet for rainbow trout without impairing fish welfare.

## 2. Materials and Methods

### 2.1. Experimental Diets

A full-fat *H. illucens* prepupae meal (H) was purchased from the Smart Bugs s.s. (Ponzano Veneto, Italy) company and stored at −20 °C in zip-lock bags until use. No information was provided by the producer on the composition of different vegetable substrate used for larval rearing, as it was considered confidential. Frozen prepupae were grinded with Retsch Centrifugal Grinding Mill ZM 1000 (Retsch GmbH, Haan, Germany) and immediately used to prepare experimental diets. A basal diet (H0) containing fish meal, pea protein concentrate, and wheat gluten meal was formulated to obtain 40 g 100 g^−1^ crude protein (CP), 18.5 g 100 g^−1^ ether extract (EE), and 22 MJ kg^−1^ gross energy (GE) in order to meet the nutrient requirements of rainbow trout [43]. The other isonitrogenous, isolipidic, and isoenergetic experimental diets were prepared by including graded levels of insect meal (25% and 50%, referred to as H25 and H50, respectively) in the H0 formulation. The experimental feeds were prepared at the Department of Agricultural, Food, Environmental and Animal Science (Di4A) of the University of Udine (Udine, Italy). All the ground ingredients (0.5 mm) and fish and vegetable oils were thoroughly blended (Kenwood kMix KMX53 stand Mixer; Kenwood, De’Longhi S.p.a., Treviso, Italy) for 20 min and water was then added to the mixture to attain appropriate consistency for pelleting. Pellets were obtained by using a 4.5-mm die meat grinder and dried at 40 °C for 48–72 h. The obtained diets were subsequently stored in under vacuum bags and kept at −20 °C until used. Diet formulation and proximate composition are shown in Table 1.

### 2.2. Chemical Composition of Feeds

The proximate composition and energy level of the H prepupae meal and of the experimental diets are shown in Table 1. Feed samples were analysed for moisture (AOAC #950.46), crude protein, CP (AOAC #976.05), ash (AOAC #920.153), and ether extract, (EE; AOAC #991.36) contents according to AOAC International [44]. The gross energy content (GE) was determined using an adiabatic calorimetric bomb (IKA C7000, Werke GmbH & Co., Staufen, Germany).

The total lipid fraction of the H prepupae meal and of the three test diets was extracted using chloroform-methanol (2:1 v:v) (Merck KGaA, Darmstadt, Germany) mixture [45]. The fatty acid methyl esters (FAMEs) were obtained following the protocol described in Morrison and Smith [46] and quantified by gas chromatography (Varian 430-GC, FID) according to Tulli et al. [47] using tricosanoic acid (C23:0; Supelco, Bellefonte, PA, USA) as an internal standard.

The grading inclusion level of H meal resulted in an increase of total saturated fatty acids (SFA) and a decrease in total polyunsaturated fatty acid (PUFA) percentage in the experimental diets as shown in Table 1.

### 2.3. Fish Rearing Conditions

The fish feeding trial was conducted at the Experimental Facility of the Di4A (Pagnacco, Udine, Italy; code 068UD047). The experimental protocol was designed according to the guidelines of the current European and Italian laws on the care and use of experimental animals (Directive 2010/63/EU, recognized in Italian legislation (D.L. 116/92)) and approved by the University of Udine Ethical Committee (Prot. N. 1/2018). All the handling procedures and sampling methods used in this trial followed the guidelines of the European Union directive 2010/63/EU on the protection of animals used for scientific purposes.

Two-hundred-and-seventy juvenile rainbow trout (*Oncorhynchus mykiss*) with an initial body weight of 137.3 ± 10.5 g were randomly allocated to nine 1-m^3^ square fiberglass tanks (30 specimens/tank). Tanks were connected to an open flow-through artesian well water system ensuring an approximate constant temperature of 13 °C, known to be near the thermal optimum for rainbow trout rearing. After stocking, fish were fed a commercial diet and were given two weeks to adapt to the experimental conditions. At the end of this period, the nine tanks were assigned to the three experimental diets (H0, H25, and H50) according to a random design with triplicate groups (tanks) per treatment. Fish were hand-fed the experimental diets over 98 days, in one daily meal (09:00 h) at 1.3% ratio of the total biomass, according to Stadtlander et al. [48]. Every three weeks, fish were group/tank weighed to adjust feeding rations. During the feeding trial, water quality parameters were monitored and recorded: temperature 12.8 ± 0.6 °C, dissolved oxygen 8.9 ± 0.43 mg L^−1^, pH 7.8 ± 0.2, total ammonia nitrogen 0.13 ± 0.02 mg L^−1^, nitrite-nitrogen < 0.015 mg L^−1^, phosphorus 1.08 ± 0.57 mg L^−1^.

### 2.4. Tissue Sampling and Calculations

At the end of the feeding trial, after a 10-h fasting period, all fish were subjected to stage 3 anaesthesia with MSS-222 (300 mg L^−1^). Biometry measurements (standard length, cm and body weight, g) and growth performance indexes such as Fulton’s condition factor (K), specific growth rate (SGR), weight gain (WG), and feed conversion ratio (FCR) were calculated per each fish/tank as follows:

K = (fish weight (g)/fish standard length^3^) × 100

SGR % = 100 × ((ln final body weight − ln initial body weight)/number of feeding days))

WG % = 100 × ((final body weight − initial body weight)/initial body weight))

FCR = total feed consumed (g) per tank biomass/weight gained (g) per tank biomass

Three fish per tank (9 fish per dietary treatment) were sacrificed by an overdose of the same anaesthetic and blood samples (approximatively 2 mL) were immediately withdrawn from caudal veins by heparinised syringes, stored on ice and centrifuged at 1500× *g* for 15 min at 4 °C. The obtained plasma was stored at −80 °C for subsequent metabolic parameter determinations. After blood sampling, liver (Li.) and digestive tract were immediately excised and washed with a 0.9% saline solution to remove the content. The digestive tract was quickly divided into medium intestine (M.I., corresponding to the tract immediately behind the anterior segment to the ileorectal valve) and hind intestine (H.I., from the ileorectal valve to the terminal part, excluding the rectum). Collected tissue (Li., M.I. and H.I.) were immediately put in individual plastic tubes, frozen in liquid N and stored at −80 °C for growth, stress, and inflammatory gene expression analyses. In addition, subsamples of the same tissues were also quickly fixed in Bouin solution (Merk Sigma Aldrich, Milan, Italy) for histological analysis or frozen on dry ice (only Li.) for subsequent FTIRI analysis.

### 2.5. Plasma Metabolic Parameters

The plasma cholesterol (Chol, mg dL^−1^), triglycerides (Trig, mg dL^−1^), glucose (Glu, mg dL^−1^), total proteins (TP, g dL^−1^), and albumin (Alb, g dL^−1^) contents were determined using an automated analyser system for blood biochemistry (Roche Cobas Mira, Biosys, Milan, Italy) and commercially available kits (Biochemical Enterprise, Milan, Italy), following the manufacturer’s protocols.

### 2.6. RNA Extraction and cDNA Synthesis

Total RNA was extracted from liver (L) and intestine samples (M.I. and H.I., approximately 90 mg) using RNAzol^®^RT reagent (Sigma-Aldrich^®^, R4533, Milan, Italy) and following the manufacturer’s instructions. RNA concentration and integrity were analysed using NanoPhotometer^®^ P-Class (Implen, Munich, Germany) and GelRed™ staining of 28S and 18S ribosomal RNA bands on 1% agarose gel, respectively. After extraction, complementary DNA (cDNA) was synthesised from 3 μg of total RNA with the High Capacity cDNA Reverse Transcription Kit (Bio-Rad, Milan, Italy), following the manufacturer’s instructions, diluted 1:10 in RNase-DNase free water and stored at −20 °C until quantitative real-time PCR (qPCR). An aliquot of cDNA was used to check primer pair specificity.

### 2.7. Real Time PCR

PCRs were performed in an iQ5 iCycler thermal cycler (Bio-Rad, CA, USA) and each sample was analysed via RT-qPCR in triplicate. Reactions were set on a 96-well plate by mixing, for each sample, 1 μL cDNA diluted 1:20, 5 μL of 2×concentrated iQ™ Sybr Green (Bio-Rad, CA, USA) as the fluorescent intercalating agent, 0.3 μM forward primer, and 0.3 μM reverse primer. The thermal profile for all reactions was 3 min at 95 °C, followed by 45 cycles of 20 s at 95 °C, 20 s at 60 °C, and 20 s at 72 °C. Fluorescent signal were detected at the end of each cycle and the melting curve analysis was performed to confirm that only one PCR product was present in these reactions. Relative quantification of the expression of genes involved in fish growth (insulin-like growth factor, *igf1,* and myostatin, *mstn1a*) and stress response (glucocorticoid receptor, *gr,* and 70-heat-shock protein, *hsp70*) in liver and inflammatory/immune response (interleukin 10, *il-10*, tumour necrosis factor, *tnf-α,* and toll-like receptor 5, *tlr-5*) in intestine was performed using *β-actin* and 60S ribosomal RNA (*60S*) as housekeeping genes to standardize the results. The primers sequences were retrieved from NCBI (http://www.ncbi.nlm.nih.gov/) and are summarised in Table 2. Amplification products were sequenced, and homology was verified. Negative controls revealed no amplification product and no primer-dimer formation was found in control templates. Data were analysed using the iQ5 optical system software version 2.0, including Genex Macro iQ5 Conversion and Genex Macro iQ5 files (all from Bio-Rad). Modification of gene expression was reported with respect to controls. Primers were used at a final concentration of 10 pmol μL^−1^.

### 2.8. Histology

Liver (Li.) and intestine (M.I., H.I.) samples were fixed by immersion in Bouin solution and stored at 4 °C for 24 h. Subsequently, samples were washed three times with 70% ethanol for 10 min and preserved in a new 70% ethanol solution. After dehydration by graded ethanol series, samples were washed with the clearing agent “Histo-Clear” (Bio-Clear, Bio-Optica, Milan, Italy) and embedded in paraffin (Bio-Optica, Milan, Italy). Paraffin blocks were cut with a microtome (Leica RM2125 RTS, GmbH, Wetzlar, Germany) and 5-μm sections were stained with Mayer haematoxylin and eosin Y (Sigma-Aldrich, Milan, Italy) and *PAS* (periodic acid of Schiff), following the manufacturer’s instructions (Bio-Optica, Milan, Italy). Stained sections were examined under a Zeiss Axio Imager.A2 (Zeiss, Oberkochen, Germany) microscope and the images were acquired by means of a combined colour digital camera Axiocam 503 (Zeiss, Oberkochen, Germany). For the quantitative image analysis of intestinal folds morphometric evaluations, ten transversal sections of M.I. at 200 μm intervals, for each fish sample were analysed. This interval was chosen in order to avoid repetitions in the measurements of intestinal folds. All the undamaged and non-oblique folds (at least 150 measurements per fish) were measured using ZEN 2.3 software (Carl Zeiss Microscopy GmbH), and the measurements were reported as means ± standard deviation (SD).

### 2.9. Fourier Transform Infrared Imaging Spectroscopy (FTIRI) Measurements and Data Analysis

FTIRI spectroscopy has been recently optimized in order to study different topics in fish species [32,49] and the protocol described by Giorgini et al. [50] was adopted to analyse liver samples of the H0, H25, and H50 trout groups. A cryotome was used to cut, from each liver sample, three thin sections (≈10 μm thickness) at 200 μm away from each other. No fixative was used. Sections were immediately deposited onto CaF_2_ optical windows (1-mm thickness, 13-mm diameter) and air-dried for 30 min. All sections were analysed at the IR beamline SISSI, ELETTRA—Synchrotron (Trieste, Italy), within 48 h after cutting. A Bruker VERTEX 70S interferometer coupled with a Hyperion 3000 Vis-IR microscope and equipped with a liquid nitrogen-cooled two-dimensional FPA detector (detector area size 164 × 164 μm^2^, 64 × 64 pixels, pixel resolution 2.56 μm; Bruker Optics GmbH, Ettlingen Germany) was used. A 15× condenser/objective was employed to obtain the visible image on each section to identify the areas of interest. IR maps (164 × 164 μm^2^) were acquired in transmission mode in the spectral range 4000–700 cm^−1^ (4096 spectra; 256 scans; spectral resolution 4 cm^−1^). Background spectra were acquired on clean portions of CaF_2_ windows. Raw IR maps were submitted to the following pre-processing treatments: (1) Atmospheric Compensation routine (OPUS 7.1 software package, Bruker Optics GmbH, Ettlingen Germany) to correct the atmospheric contributions of carbon dioxide and water vapour, and (2) vector normalization in the 3800–950 cm^−1^ spectral range to avoid artefacts induced by local thickness variations (OPUS 7.1 software package). Pre-processed IR maps were then integrated under the following spectral ranges, to rebuild the topographical distribution of lipids, proteins and glycogen: 3000–2825 cm^−1^ (overall lipids, *Lipids*); 1720–1480 cm^−1^ (overall proteins, *Proteins*); 1180–1000 cm^−1^ (overall glycogen, *Glycogen*). An arbitrary colour scale was used with warm (red) to white colour indicating the highest absorbance value, while blue colour represented the lowest.

On all the spectra of each IR map, several bands with biological relevance were selected and the corresponding integrated areas calculated by using the Integration routine (OPUS 7.1): 3000–2825 cm^−1^ (asymmetric and symmetric stretching modes of CH_2_ and CH_3_ groups mainly in lipid alkyl chains, LIP); 2943–2895 cm^−1^ (asymmetric stretching mode of CH_2_ groups in lipid alkyl chains, CH2); 1711–1483 cm^−1^ (Amide I and II bands of proteins, PRT); 1181–1142 cm^−1^ (stretching of C-O-H groups in carbohydrates, COH); 1073–1000 cm^−1^ (stretching of C-O and C-C moieties and bending of C-O-H groups in carbohydrates, mainly attributed to glycogen, GLY). The integrated areas of the spectral regions at 3000–2825 cm^−1^ and 1767–950 cm^−1^ were added and taken as representative of the overall tissue biomass (TBM). The following band area ratios were then calculated: LIP/TBM (total amount of lipids); CH2/TBM (total amount of saturated alkyl chains); CH2/LIP (saturated alkyl chains with respect to total lipids); PRT/TBM (total amount of proteins); GLY/TBM (total amount of glycogen), and COH/TBM (total amount of carbohydrates).

The average IR absorbance spectra of H0, H25 and H50 liver trout groups were also calculated in the spectral range from 4000 to 900 cm^−1^ and converted to second derivative mode, to obtain the position of the most meaningful absorption bands.

### 2.10. Statistical Analyses

Data are presented as mean value ± SD. Data were tested for normality and homogeneity of variances by using Shapiro–Wilk’s and Levene’s tests, respectively. One-way ANOVA was adopted for growth parameters where the tank (fish group) was the experimental unit. In case of plasma metabolites, where the individual fish was the experimental unit, data analysis was carried out using a mixed ANOVA model, including the tank as a random variable. When appropriate, the Tukey’s post hoc test (significant level *p* < 0.05) to detect significant differences among the dietary treatments was used. All analyses were performed by using the SPSS package (SPSS Inc., Chicago, IL, USA). For the gene expression and FTIRI results, the statistical software package Prism5 (GraphPad software, company, city, state abbrev if USA, country)) was used and one-way analysis of variance was performed followed by Student’s test for the comparison of M.I. and H.I. results.

## 3. Results

### 3.1. Fish Growth

Fish growth performance parameters are reported in Table 3. The survival rate of fish was 100% over 98 days of feeding trial for all the experimental groups. The fish readily accepted the experimental diets and all feeds were consumed without rejection or loss. Fish fed both diets containing insects (H25 and H50) resulted in a final body weight (FBW), K, WG, SGR and FCR that were not significantly different (*p* > 0.05) from those attained by fish fed the H0 diet.

### 3.2. Plasma Metabolic Parameters

The plasma metabolic parameters obtained from samples collected at the end of feeding trial are reported in Table 4. Plasma cholesterol (Chol), triglycerides (Trig), glucose (Glu), total proteins (TP), and albumin (Alb) levels did not show significant differences among the dietary groups (*p* > 0.05).

### 3.3. Gene Expression

In liver samples, at the end of feeding trial, real-time PCR analyses were performed on genes involved in fish growth (*igf1* and *mstn1a*) and stress response (*gr*, *hsp70*).

**Figure 1:**
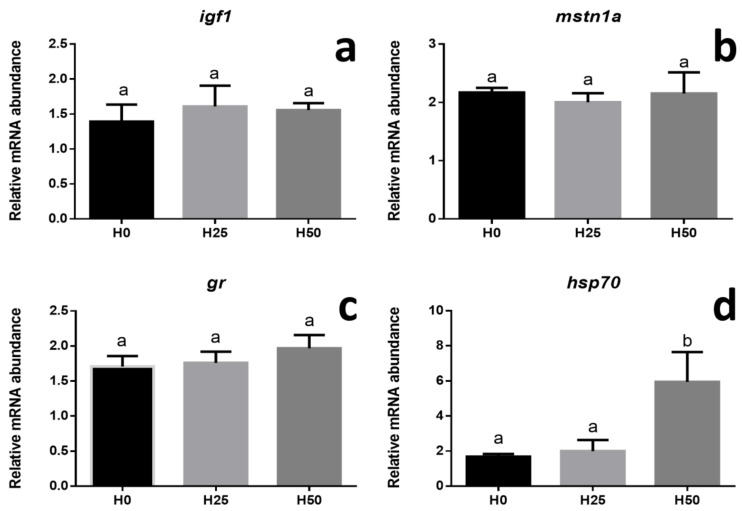
Relative mRNA abundance of genes involved in fish growth (*igf1* and *mstn1a*) and stress (*gr* and *hsp70*) analysed in liver from trout fed diets including different H meal levels (H0, H25, H50). (**a**) *igf1*. (**b**) *mastn1a.* (**c**) *gr*. (**d**) *hsp70*. Different letters indicate significant differences among the experimental groups (*p* <0.05). Values are presented as mean ± SD.

Growth biomarkers, *igf1* and *mstn1a* (Figure 1a,b), did not show significant differences (*p* > 0.05) among the dietary treatments. The expression of the genes involved in stress response (*gr* and *hsp70*), *gr* did not show any significant difference among the experimental groups (Figure 1c), while *hsp70* showed a significant upregulation only in H50 dietary treatment (*p* < 0.05) (Figure 1d).

**Figure 2:**
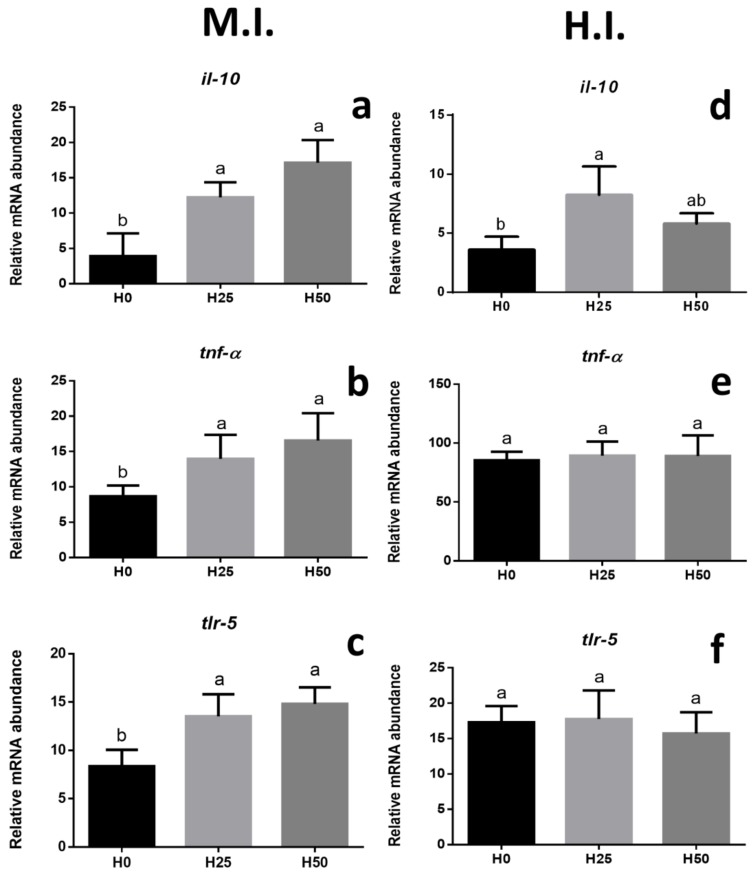
Relative mRNA abundance of genes involved in inflammatory response and innate immune defence, analysed in the medium (M.I.) and hind (H.I.) intestine. (**a**) M.I. *il-10*. (**b**) M.I. *tnf-α*. (**c**) M.I. *tlr-5*. (**d**) H.I. *il-10*. (**e**) H.I. *tnf-α.* (**f**) M.I. *tlr-5*. Different letters indicate statistically significant differences among experimental groups (*p* < 0.05). Values are presented as mean ± SD.

The inflammatory response was investigated in the medium (M.I.) and hind (H.I.) intestine through the gene expression of cytokines (*il-10*, *tnf-α*), while the innate immune defence was analysed by monitoring the membrane receptor *tlr-5*. The most significant differences were shown in the M.I. (Figure 2). In particular, a significant (*p* < 0.05) higher gene expression of *il-10* (Figure 2a), *tnf-α* (Figure 2b), and *tlr-5* (Figure 2c) was shown in both H25 and H50 groups compared to H0 group. As regards the expression of the same genes in the H.I., no significant differences were observed among the three experimental groups, except for a higher *il-10* gene expression in the H25 group (Figure 2d).

### 3.4. Histology

**Figure 3:**
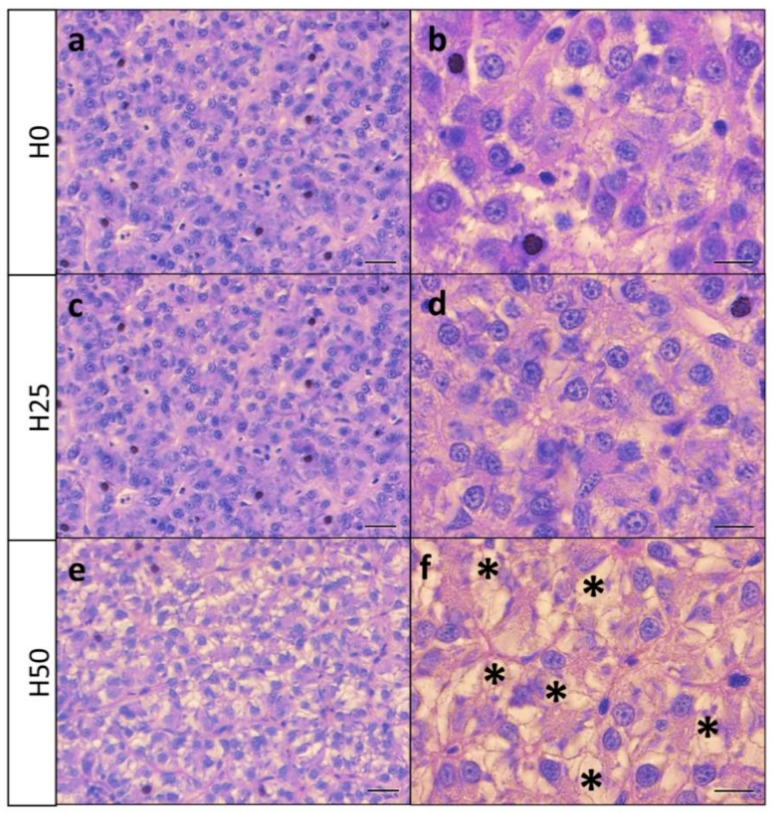
Liver histology of rainbow trout stained with periodic acid of Shiff (PAS). Low and high magnification of sections of liver from group H0 (**a**,**b**), H25 (**c**,**d**) and H50 (**e**,**f**). (Asterisks indicate lipid accumulation. Scale bars: (**a**,**c**,**e**) = 20 μm; (**b**,**d**,**f**) = 10 μm.

Histological analysis of the liver stained with periodic acid of Shiff (PAS) is reported in Figure 3. H50 (Figure 3e,f) showed an increase in liver lipid accumulation compared to both H0 (Figure 3a,b) and H25 (Figure 3c,d). No appreciable differences in glycogen accumulation were observed between the dietary treatments by means of PAS staining.

**Figure 4:**
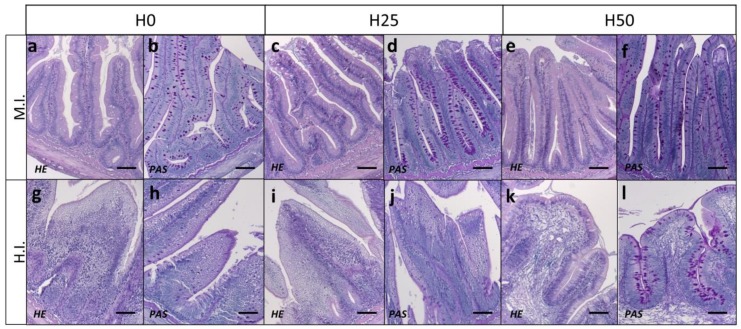
Histology of medium (M.I.) and hind (H.I.) intestine from the different feeding groups (H0, H25 and H50). Section were stained with haematoxylin and eosin (HE) and periodic acid of Shiff (*PAS*). (**a**) H0 in M.I. HE staining. (**b**) H0 in M.I. *PAS* staining. (**c**) H25 in M.I. HE staining. (**d**) H25 in M.I. *PAS* staining. (**e**) H50 in M.I. HE staining. (**f**) H50 in M.I. *PAS* staining. (**g**) H0 in H.I. HE staining. (**h**) H0 in H.I. *PAS* staining. (**i**) H25 in H.I. HE staining. (**j**) H25 in H.I. *PAS* staining. (**k**) H50 in H.I. HE staining. (**l**) H50 in H.I. *PAS* staining. Arrow heads indicate mucous cell hypertrophy. Scale bar = 100 μm.

Concerning the histological analysis of the different intestinal tracts (M.I. and H.I.), conventional haematoxylin and eosin staining (HE) was performed to investigate possible inflammatory alterations, while periodic acid of Shiff staining (*PAS*) was used to highlight differences in mucous cells distribution (Figure 4). No inflammatory events were shown in both M.I. and H.I. of all fish examined (Figure 4a,c,e,g,i,m). On the contrary, an appreciable increase in mucous cells (hyperplasia) was shown in the H.I. tract of fish fed H50 (Figure 4n) compared to both H0 and H25.

Finally, the morphometric evaluation of the intestinal fold length was performed in the medium tract of the intestine (M.I.) to evaluate a possible reduction of the absorptive epithelial surface. Measurements evidenced a significant shortening of the fold length in fish fed diets containing insects (697.5 ± 15.9 µm and 681.1 ± 11.2 µ for H25 and H50, respectively) compared to fish fed the control diet H0 (821.7 ± 36.8 µm) (*p* < 0.05).

### 3.5. FTIRI Analysis

**Figure 5:**
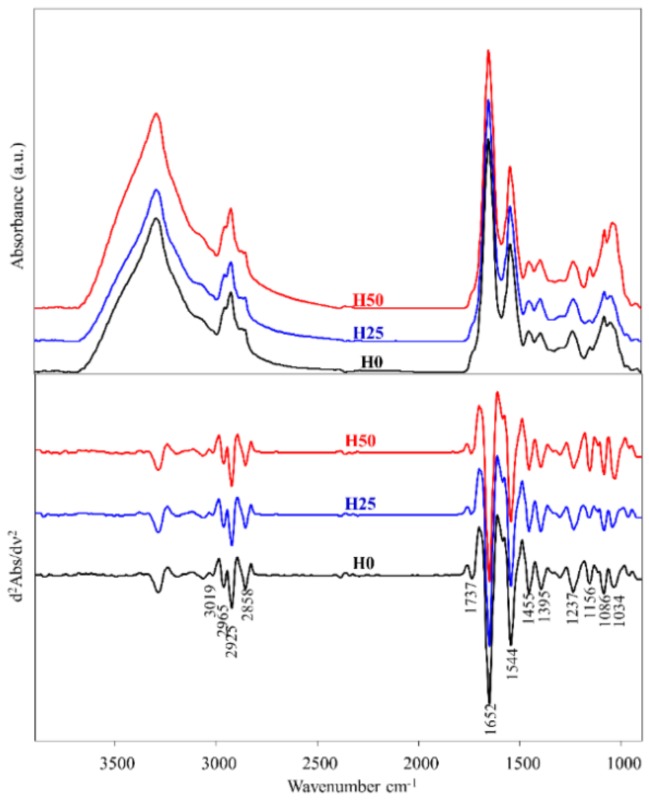
Average spectra of liver sections of rainbow trout fed the test diets: H0 (black), H25 (blue), and H50 (red). Spectra were reported in the 4000–900 cm^−1^ spectral range in absorbance and second derivative modes (the wavenumbers of the most relevant peaks are reported in the bottom part).

In Figure 5, the average spectra of liver sections of all experimental groups were reported in absorbance and second derivative modes. In Table 5, the most relevant absorption bands are listed together with the vibrational meaning and the biochemical assignment, which were done according to the literature [35,48]. Modifications in the spectral profiles were detected mainly in the regions associated with the vibrational modes of lipid alkyl chains (3000–2824 cm^−1^) and carbohydrates (1180–1000 cm^−1^).

**Figure 6:**
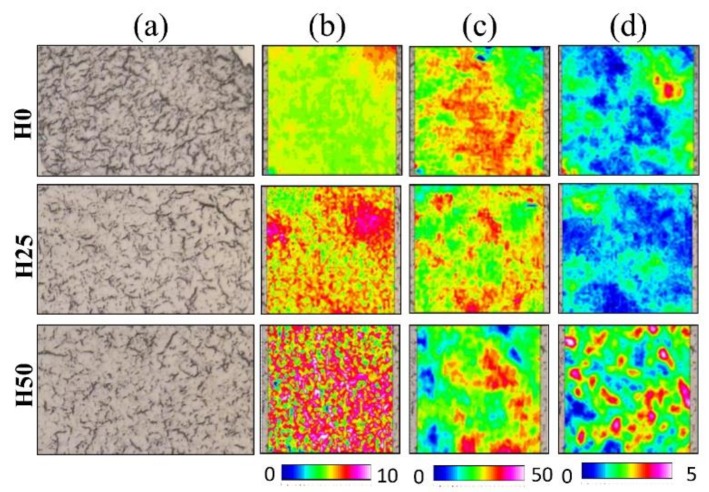
Microphotographs (**a**) of representative liver sections of H0, H25, and H50 groups analysed by FTIRI and topographical distribution of *Lipids* (**b**), *Proteins* (**c**), and *Glycogen* (**d**). Colours from warm (red) to white indicate higher absorbance values, whilst blue colour indicates the lower ones. See colour scale at the bottom.

A preliminary imaging analysis was performed on IR maps to retrieve information on the topographical distribution and the relative amount of *Lipids* (Figure 6b), *Proteins* (Figure 6c), and *Glycogen* (Figure 6d) in liver sections from the different experimental groups. H50 liver samples exhibited higher amounts of *Lipid* and *Glycogen*, together with a moderate decrease of *Proteins* than liver of fish fed H0; in H25, only an increase in total *Lipids* was detected compared to H0 (Figure 6b), while *Proteins* and *Glycogen* showed similar amounts.

**Figure 7:**
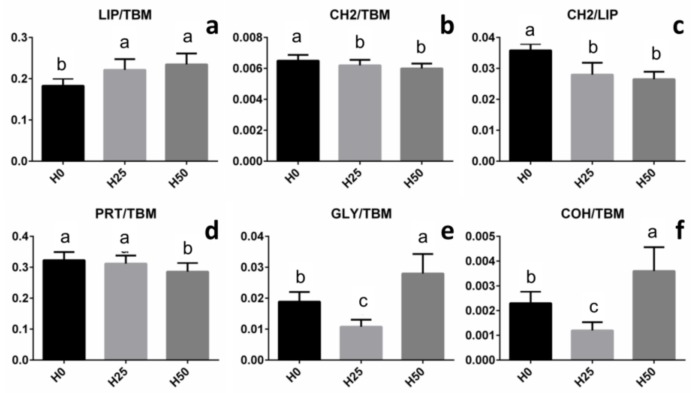
Statistical analysis of band area ratios calculated on H0, H25, and H50 liver samples: (**a**) LIP/TBM (total amount of lipids), (**b**) CH2/TBM (total amount of saturated alkyl chains), (**c**) CH2/LIP (saturated alkyl chains with respect to total lipids), (**d**) PRT/TBM (total amount of proteins), (**e**) GLY/TBM (total amount of glycogen), and (**f**) COH/TBM (total amount of carbohydrates). Values are presented as mean ± SD. Different letters indicate statistically significant differences among the experimental groups (*p* < 0.05).

The semi-quantitative analysis performed on specific band area ratios allows for making the following considerations (Figure 7): (1) the relative amount of lipids calculated on tissue biomass (LIP/TBM) resulted in significantly increased H25 and H50 compared to H0 (*p* < 0.05) (Figure 7a); (2) the relative amounts of saturated lipid alkyl chains calculated both on tissue biomass (CH2/TBM) and on total lipids (CH2/LIP) were significantly lower in H25 and H50 compared to H0 (*p* < 0.05) (Figure 7b,c); (3) a significant decrease of the relative amount of proteins calculated on tissue biomass (PRT/TBM) was detected only in H50, with respect to H0 and H25 (*p* < 0.05) (Figure 7d); and (4) the highest levels of glycogen and carbohydrates, calculated on tissue biomass (GLY/TBM and COH/TBM, respectively), were found in H50, and the lowest in H25 (*p* < 0.05) (Figure 7e,f).

## 4. Discussion

In the present study, inclusion of full-fat *Hermetia illucens* meal in practical diets for rainbow trout resulted in a moderate but not significant reduction of growth and FCR. These findings are in agreement with several previous studies which tested inclusion levels (≤50%) of black soldier fly (BSF) in the same [21,48] and different fish species, such as channel catfish (*Ictalurus punctatus*) [51], Atlantic salmon (*Salmo salar*) [26,52], Jian carp (*Cyprinus carpio*) [36], turbot (*Psetta maxima*) [14], gilthead seabream (*Sparus aurata*) [53], European sea bass (*Dicentrarchus labrax*) [24], Nile tilapia (*Oreochromis niloticus*) [54], and yellow catfish (*Pelteobagrus fulvidraco*) [55].

Nevertheless, results on full-fat or defatted *Hermetia* meal inclusion in aquafeeds are still contradictory. For example, in rainbow trout, dietary inclusion level of 30% or 33% of full-fat *Hermetia* prepupae meal caused a worsening in WG and FCR values [19,20]. Similarly, an inverse relationship was observed in trout fed increasing dietary amount of defatted BSF meal both on thermal-unit growth coefficient and FCR [56]; this was also evidenced in juvenile turbot [14]. These contradictory results may be related to several factors such as the BSF dietary inclusion level, the use of full-fat or defatted insect meal, the presence of chitin, the feeding regime to which fish were subjected (restricted vs. apparent satiation), the manufacturing of the feeds (pelleting vs. extrusion) and, of course, the fish species investigated as well as the stage of development (juvenile vs. adult). Therefore, further studies are needed to better elucidate the physiological responses of fish to these new diets, including the species-specificity of these responses.

Since growth and ontogeny follow a genetically programmed and well-defined sequence in which gene transcription and hormone regulation are crucial, clinical and zootechnical parameters may not be sufficient to monitor fish growth and development. Therefore, besides the traditional markers (morphological, histological, physiological, and biochemical), it may be important to look for alternative ones at the molecular level. Consequently, gene expression can be used to generate useful insights linking biotic and abiotic conditions to individual performances. Molecular markers can be identified among those genes whose expression could reasonably be modified by different conditions, including nutrition [57,58] and, among these, several studies showed that *igfs* and *mstn* are useful growth biomarkers. Nutritional deficiencies have deep impact on fish growth and welfare and circulating IGFs levels are known to be nutritionally regulated [33,35,59,60]. In fact, in many fish species, IGFs blood or tissue levels mRNA positively correlate with feeding levels, dietary protein content and body growth rate [59]. On the other hand, myostatin, a member of the transforming growth factor-β (TGF-β) family, is considered to be an inhibitory factor in the growth of skeletal muscle [61]. Therefore, fish final growth is related to the interplay of these positive and negative signals [62]. In the present study, biometric results were fully supported by the molecular ones since no significant differences in *igf1* and *mstn1a* gene expression were detected among the experimental groups.

Captive rearing, including nutrition, is quite different from a natural condition, often causing a decline in fish welfare. The Fulton’s condition factor (K), commonly used to describe fish well-being, is based on the assumption that for a given length, heavier fish are in better condition [63]. K values lower than 1 indicate unhealthy fish, while values higher than 1 denote fish in a good physiological state. Independent of dietary treatment, the K values reported in the present trial were higher than 1 and similar to those recorded in a previous study carried out in adult rainbow trout fed defatted *Hermetia* meal [21]. Farmed fish are usually exposed to a variety of stressors and long-term exposure has negative effects on fish health and performance, by increasing disease susceptibility and decreasing fish growth [64]. However, to our knowledge, scant information is available for rainbow trout regarding the effects of insect meal feeding on the expression of stress and immune-related genes, as well as on the levels of serum metabolites as secondary and tertiary stress responses, respectively [65].

The results of the present study have demonstrated that a 98-day feeding trial with graded dietary inclusion level of full-fat insect meal had no significant effects on the activation of the primary stress response (*gr*) gene expression in trout. At a cellular level, the stress response is often mediated by heat-shock proteins (HSPs), a family of highly conserved proteins present in all cells and life forms [66]. A variation in HSPs gene expression is often considered a useful bio-indicator in stress response [67].

An up-regulation of the *hsp70* gene expression was observed, at the hepatocyte level, in fish fed the highest insect meal dietary inclusion (H50) possibly suggesting a physiological activation of stress/inflammation response after 98 days of feeding trial. Similarly, a significant up-regulation of *hsp70* was observed in Jian carp fed a diet including defatted BSF larvae higher than 79 g kg^−1^, suggesting a potential stress response induction [36]. On the contrary, in Atlantic salmon [68] *hsp70* gene expression was not affected by the inclusion of 600 g kg^−1^ of defatted insect meal. The present contradictory results obtained in these trailblazing studies deserve further investigation in order to clarify the stress response of fish fed diets including insect meal. Anyway, considering the plasma metabolic profile of trout fed graded inclusion level of full-fat *Hermetia* meal, no significant differences were noted in the parameters herein considered, which were in line with those previously found in the same species [69]. The inconsistency of our results and those observed in other fish species, such as European seabass [24], deserves to be further investigated in order to better clarify the potential ipo-cholesterolaemic effect of insect meal.

As a primary site of food digestion and nutrient absorption, the gastrointestinal system plays a key role in the optimum utilization of dietary nutrients, which depends on its functionality. Previous studies on alternative feed ingredients show that high inclusion levels of plant protein as FM replacer can affect both gut and liver integrity [70]. Liver plays a key role in many fish metabolic pathways and its morphological structure, macromolecular composition and gene expression are deeply influenced by the diet [33,34,35]. In this regard, the present study, demonstrates that stress biomarkers are affected by diets including insects. Even if the offered diets were isolipidic, the lipid hepatic accumulation detected by histological and FTIRI analyses was very different. Specifically, fish fed diets including insect meal showed an increase in lipid accumulation compared to control fish (*Lipids* and LIP/TBM). FTIRI analysis provided deeper insight into liver biochemical composition, revealing that most of the accumulated lipids showed shorter alkyl chains (CH2/TBM and CH2/LIP). In addition, a high accumulation of glycogen (*Glycogen* and GLY/TBM) and, in general, of carbohydrates (COH/TBM), was detected in trout liver fed the diet with the highest insect meal inclusion. Finally, no relevant changes in the protein pattern (*Proteins* and PRT/TBM) were observed; when the highest insect meal inclusion level was used, only a moderate decrease of proteins was found.

A recent paper by Zarantoniello et al. [59] reported that an increase in the dietary SFA may play an important role in the development of hepatic steatosis [71], causing liver dysfunction by promoting endoplasmic reticulum stress and apoptosis [72,73] and these results are in line with the *hsp70* up-regulation observed in fish fed the H50 diet. Aside from the deficiencies in fatty acid composition, insect meal is characterized by the presence of chitin, a polysaccharide that constitutes the insect and crustacean exoskeleton. The specific role of chitin in fish diets is still controversial and is related to its dietary level of inclusion; when included at low levels, it might act as an immune-stimulant and anti-inflammatory molecule in fish [74], while if included at high doses it might reduce fish growth and intestinal inflammation [14,36]. In addition, the role of chitin seems to be size dependent: large chitin polymers may be inert; fragments of 40–70 μm may be pro-inflammatory and the smaller ones (less than <40 μm) anti-inflammatory [75]. Consequently, when using diets including chitin, the gastrointestinal tract should be carefully analysed, possibly through a multidisciplinary approach.

Intestinal morphology is a valuable means to assess both gut health and its functional *status* [32]. In the present study, the histological analyses performed in the medium and hind intestine highlighted the absence of severe inflammatory events but evidenced the presence of goblet cells that produce neutral mucins, as previously observed in trout fed defatted insect meal [27,76] and in black tetra (*Gymnocorymbus ternetzi*) [77]. Mucus-secreting cells are normally observed in the mucosa of digestive tract of fish, with distribution and histochemical characterization depending on species, age, gut segment, diets and feeding habits [27]. In the present study, the mucin type cells were studied along the intestinal tract [76] and an increasing amount of mucin goblet cells were specifically observed in the hind gut tract of fish fed the highest *Hermetia* meal inclusion (H50). These results are in agreement with previous observations in trout [27], tiger barb (*Puntius tetrazona*), black tetra [77], and in rice field eel (*Monopoterus albus*) [78], in which the accumulation of mucin cells in the posterior intestine was associated with intestinal protection and lubrification [77]. Dietary manipulation is also known to influence the intestinal microvilli structure [79] and, in order to assess possible gut histopathological evidences, villi length was investigated [36,58,79]. Since proteins are mainly digested and absorbed in the proximal and mid intestine, and to a lesser extent in the hind tract (H.I.), the medium portion of the intestine (M.I.) was analysed. The present study evidenced a negative effect on M.I. villus length in fish fed insect including diets showing both a reduction in the absorptive epithelial surface and a potential occurrence of gut inflammation [36,37]. These histological observations were fully supported by the gene expression analyses (which can be useful to precociously detect physiological responses) of the immune related genes [80], showing a significantly higher gene expression in M.I. respect to the other intestinal tracts analysed. Similar negative effects were previously observed in Jian carp [36] fed a diet including defatted BSF larvae meal up to 79 g kg^−1^ resulted in irregularity of gut microvilli shape. However, other studies performed on Salmonids evidenced no significant histological modifications at intestinal level [21,26,27].

In this study, graded levels of full-fat *H. illucens* dietary meal inclusion were associated with increasing levels of purified plant protein-rich ingredients, such as pea protein concentrate and wheat gluten, in order to keep all diets isonitrogenous and isoenergetic. Even if interactive effects between protein sources of different origin cannot be ruled out [81], they were expected to be minor in magnitude, hence supporting the idea that changes in fish responses were basically due to the graded levels of insect meal in the diet. In fact, in this experiment, both pea protein concentrate and wheat gluten were included at levels that were shown not to alter growth and gut health of salmonids when used either singly or in combination [39,40,41,42].

## 5. Conclusions

In conclusion, the present study highlighted that dietary inclusion of full-fat *H. illucens* meal up to 210 g kg^−1^ in a practical diet for rainbow trout did not hamper fish growth; however, the application of a multidisciplinary approach evidenced that some biomarkers related to stress and immune function were altered, as well as liver macromolecular composition.

Further investigations are needed to deepen the knowledge of dietary manipulation and mechanisms responsible for potential combined effects when insect meal and other plant protein sources are used in practical diets for rainbow trout.

## Figures and Tables

**Table 1:** Ingredients (g kg^−1^), proximate composition (g 100 g^−1^) and summary of the major fatty acid classes (as percentage of total FAMEs) of prepupae meal (H) and the experimental diets. H0, basal diet containing conventional ingredients; H25 and H50, experimental diets containing graded inclusion levels of H meal.

Diet Formulation and Composition	H	H0	H25	H50
Ingredients				
Chile prime fish meal ^1^		420	315	210
Pea protein concentrate ^2^		55	78	100
H meal ^3^		-	105	210
Wheat gluten meal ^4^		55	78	100
Wheat flour ^5^		290	268	255
Fish oil		70	40	28
Palm oil		70	75	56
Mineral supplement ^$^		10	10	10
Vitamin supplement #		10	10	10
Binder		20	20	20
L-Methionine		-	1	1
Proximate composition *				
Moisture	20.93 ± 0.02	4.24 ± 0.03	5.49 ± 0.03	5.31 ± 0.18
Crude protein, CP	30.84 ± 0.38	40.27 ± 0.45	39.98 ± 0.37	40.16 ± 0.39
Ether extract, EE	33.10 ± 0.24	18.63 ± 0.27	18.56 ± 0.14	17.68 ± 0.20
Ash	10.30 ± 0.18	14.30 ± 0.28	14.20 ± 0.23	14.13 ± 0.31
Gross energy (MJ kg^−1^)	n.d. ^6^	22.10 ± 0.11	22.30 ± 0.03	21.28 ± 0.06
Fatty acid composition *				
SFA	65.30 ± 2.05	33.76 ± 0.14	42.13 ± 4.51	48.24 ± 1.67
MUFA	28.37 ± 0.89	32.87 ± 1.23	34.46 ± 0.61	33.04 ± 1.45
PUFA	6.34 ± 0.41	33.00 ± 1.41	23.00 ± 1.14	18.50 ± 2.12
PUFA n-3	0.66 ± 0.04	22.01 ± 0.45	13.71 ± 0.22	10.11 ± 0.33
PUFA n-6	5.68 ± 0.21	11.33 ± 2.20	9.68 ± 1.56	8.62 ± 1.50
n-9	18.07 ± 1.14	26.50 ± 0.71	28.00 ± 1.41	25.50 ± 2.12
EPA	0.20 ± 0.02	6.85 ± 0.24	4.24 ± 0.49	3.12 ± 0.74
DHA	-	13.42 ± 0.89	8.24 ± 0.36	5.97 ± 0.72
n-3/n-6	0.12 ± 0.10	1.99 ± 0.43	1.44 ± 0.25	1.19 ± 0.17

^1^ Bioceval GmbH & Co. KG Cuxhaven, Germany; ^2^ Lombarda trading srl, Casalbuttano & Uniti (CR, Italy); ^3^ SmartBugs srl (Treviso, Italy); ^4^ Sacchetto spa (Torino, Italy); ^5^ Consorzio Agrario (Pordenone, Italy); ^$^ Mineral supplement composition (% mix): CaHPO_4_*2H_2_O, 78.9; MgO, 2.725; KCl, 0.005; NaCl, 17.65; FeCO_3_, 0.335; ZnSO_4_.H_2_O, 0.197; MnSO_4_.H_2_O, 0.094; CuSO_4_.5H_2_O, 0.027; Na_2_SeO_3_, 0.067; # Vitamin supplement composition (% mix): thiamine hydrochloride (vitamin B1), 0.16; riboflavin (vitamin B2), 0.39; pyridoxine hydrocloride (vitamin B6), 0.21; cyanocobalamine (vitamin B12), 0.21; niacin (vitamin PP or B3), 2.12; calcium pantotenate, 0.63; folic acid, 0.10; biotin (vitamin H), 1.05; myo-inositol (vitamin B7), 3.15; stay C Roche (vitamin C), 4.51; tocopherol (vitamin E), 3.15; menadione (vitamin K3), 0.24; retinol (vitamin A 2500 UI kg^−1^ diet), 0.026; cholecalciferol (vitamin D3 2400 UI kg^−1^ diet), 0.05; choline chloride, 83.99; * Values reported as mean of triplicate analyses; ^6^ n.d.: not determined. SFA = C10:0 + C12:0 + C13:0 + C14:0 + C15:0 + C16:0 + C17:0 + C18:0 + C20:0 + C21:0 + C22:0 + C24:0. MUFA= C16:1n-9 + C16:1n-7 + C18:1n-9 + C18:1n-7 + C20:1n-9 + C22:1n-9 + C24:1n-9. PUFA= C18:2n-6 + C18:3n-3 + C18:3n-6 + C20:2n-6 + C20:3n-3 + C20:3n-6 + C20:4n-6 + C20:5n-3 + C22:6n-3. PUFA n-3 = C18:3n-3 + C20:3n-3 + C20:5n-3 + C22:6n-3. PUFA n-6 = C18:2n-6 + C18:3n-6 + C20:2n-6 + C20:3n-6 + C20:4n-6. SFA: saturated fatty acid; MUFA: monounsaturated fatty acid; EPA: eicosapentaenoic acid; DHA: docosahexaenoic acid; PUFA: polyunsaturated fatty acid; FAME: fatty acid methyl ester.

**Table 2:** Oligonucleotide primers and Annealing Temperature (A.T.) of each gene investigated in this study.

Target Tissue	Gene Name	Primer Sequence		A.T. (°C)	Gene Bank Accession Number
Liver		Forward	Reverse		
	igf1	TGGACACGCTGCAGTATGTGTGT	CACTCGTCCACAATACCACGGT	60	GQ924783
	mstn1a	CCGCCTTCACATATGCCAA	CAGAACCTGCGTCAGATGCA	60	AY839106
	gr	GCCTTTTGGCATGTACTCAAACC	GGACGACTCTCCATACCTGTTC	60	AY549305
	hsp70	CCCTGGGCATCGAAACC	CCCTCGTAGACCTGGATCATG	60	AY423555
Intestine	il-10	CGACTTTAAATCTCCCATCGA	GCATTGGACGATCTCTTTCTT	59	DQ821115
	tnf-α	AGCATGGAAGACCGTCAACGAT	AGCATGGAAGACCGTCAACGAT	60	DQ070246
	tlr-5	GGCATCAGCCTGTTGAATTT	ATGAAGAGCGAGAGCCTCAG	57	NP001118216
	β-actin	AGACCACCTTCAACTCCATCAT	AGAGGTGATCTCCTTCTGCATC	60	AJ537421
	60S	TTCCTGTCACGACATACAAAGG	GTAAGCAGAAATTGCACCATCA	60	DT044641.1

**Table 3:** Growth response of rainbow trout fed the experimental diets.

Growth Metrics	H0	H25	H50
FBW (g) ^1^	301.21 ± 32.21	279.59 ± 37.26	251.27 ± 22.14
K ^2^	1.13 ± 0.11	1.13 ± 0.01	1.12 ± 0.07
WG (%) ^3^	119.81 ± 16.83	102.91 ± 24.24	83.18 ± 14.32
SGR (%) ^4^	0.80 ± 0.08	0.71 ± 0.12	0.61 ± 0.08
FCR ^5^	1.02 ± 0.17	1.22 ± 0.35	1.47 ± 0.28

Data are reported as mean of triplicate tanks and presented as mean ± SD. ^1^ Final body weight, ^2^ Fulton’s condition factor, ^3^ Weight gain, ^4^ Specific growth rate, ^5^ Feed conversion ratio.

**Table 4:** Plasma metabolic parameters measured in rainbow trout fed the test diets at the end of the 98-day feeding period.

Plasma Parameters	H0	H25	H50
Chol (mg dL^−1^)	188.0 ± 20.2	178.6 ± 29.9	196.7 ± 40.8
Trig (mg dL^−1^)	212.4 ± 50.1	186.8 ± 55.6	202.5 ± 37.8
Glu (mg dL^−1^)	112.7 ± 16.8	103.6 ± 9.9	123.0 ± 31.1
TP (g dL^−1^)	3.5 ± 0.5	3.2 ± 0.6	3.4 ± 0.7
Alb (g dL^−1^)	1.3 ± 0.2	1.5 ± 0.4	1.6 ± 0.3

Chol: cholesterol; Trig: triglycerides; Glu: glucose; TP: total proteins, and Alb: albumin. Values are reported as mean ± SD.

**Table 5:** Absorption bands detected on second derivative IR spectra of H0, H25 and H50 experimental groups. For each band, the wavenumber (expressed as cm^−1^), together with the vibrational mode and the biological meaning are reported.

Wavenumber	Vibrational Mode	Biological Meaning
~3019	Stretching vibration of =CH groups	
~2965, ~2925, ~2858	Asymmetric and symmetric stretching vibrations of CH_3_ and CH_2_ groups	Mainly lipid alkyl chains
~1737	Stretching vibration of C=O ester moieties	
~1662, ~1544	Amide I and II bands	Proteins
~1455, ~1395	Bending vibrations of proteins side chains	
~1237, ~1087	Asymmetric and symmetric stretching vibrations of PO_2_- groups	Phosphate groups
~1156	Stretching vibrations of C-O-H moieties	Carbohydrates and glycogen
~1034	Stretching vibrations of C-O and C-C moieties and bending of C-O-H groups	
